# 2-(2,4-Di­chloro­phen­oxy)-*N*′-[2-(2,4-di­chloro­phen­oxy)acet­yl]acetohydrazide

**DOI:** 10.1107/S2414314621003187

**Published:** 2021-04-09

**Authors:** Gamal A El-Hiti, Bakr F. Abdel-Wahab, Emad Yousif, Amany S. Hegazy, Benson M. Kariuki

**Affiliations:** a Cornea Research Chair, Department of Optometry, College of Applied Medical Sciences, King Saud University, PO Box 10219, Riyadh 11433, Saudi Arabia; b Applied Organic Chemistry Department, National Research Centre, Dokki, Giza 12622, Egypt; c Department of Chemistry, College of Science, Al-Nahrain University, Baghdad 64021, Iraq; d School of Chemistry, Cardiff University, Main Building, Park Place, Cardiff CF10 3AT, UK; University of Aberdeen, Scotland

**Keywords:** crystal structure, acyl­hydrazine, biological activity

## Abstract

The complete mol­ecule of the title compound, C_16_H_12_Cl_4_N_2_O_4_, is generated by a crystallographic centre of symmetry. In the crystal, N—H⋯O hydrogen bonds link the mol­ecules into [010] chains featuring *R*_2_^2^(10) loops. The chains are cross-linked by short Cl⋯N contacts [3.224 (2) Å].

## Abstract 2

In the crystal of the title compound, the mol­ecules are linked into chains by N—H⋯O hydrogen bonds. The chains are inter­linked by short Cl⋯N contacts.

## Graphical abstract

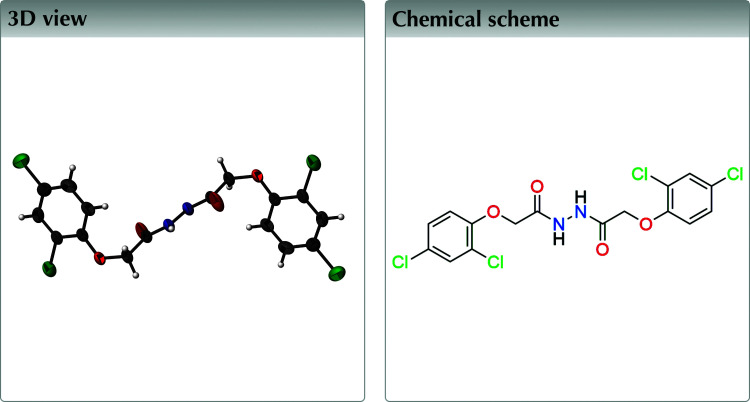

## Structure description

**Figure 1:**
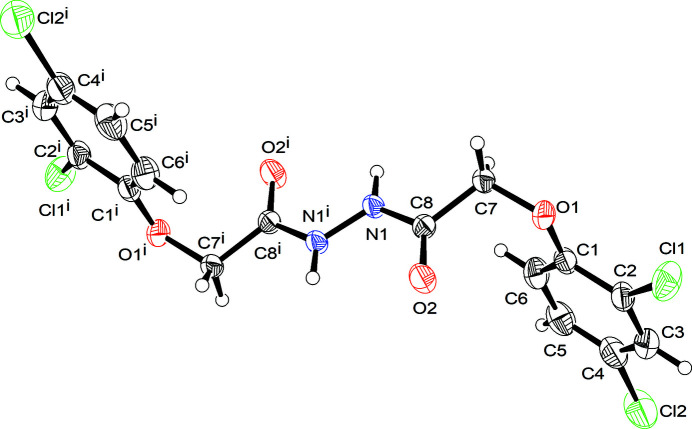
The mol­ecular structure of (**I**) showing 50% displacement ellipsoids. Symmetry code: (i) 1 − *x*, 2 − *y*, 1 − *z*.

Di­acyl­hydrazines have insecticidal activities (Wang *et al.*, 2017 ▸) and can also be used to recover metal ions from solution (Chekanova *et al.*, 2004 ▸; Radushev *et al.*, 2007 ▸). In addition, they are precursors in the synthesis of biologically active heterocycles (Zarei 2017 ▸; Stabile *et al.*, 2010 ▸). As part of our studies in this area, we now describe the synthesis and structure of the title compound, C_16_H_12_Cl_4_N_2_O_4_ (**I**). The asymmetric unit consists of half a mol­ecule, which is completed by inversion symmetry centred in the middle of the central N—N bond (Fig. 1 ▸).

The twist angle between the 2,4-di­chloro­phen­oxy ring system and the *N′*-acetyl­acetohydrazide group in (**I**) is 77.8 (1)°; the latter has a crystallographically imposed *trans* conformation, in a manner similar to 2-[5-methyl-2-(propan-2-yl)phen­oxy]-*N′*-{2-[5-methyl-2-(propan-2-yl)phen­oxy]acet­yl}acetohydrazide (Fun *et al.*, 2011 ▸). The C—N—N—C torsion angles in the structures of 2-(4-chloro­phen­oxy)-*N′*-[2-(4-chloro­phen­oxy)acet­yl]acetohydrazide monohydrate (Chen & Tan, 2010 ▸) and *N,N′*-*bis*[2-(quinolin-8-yl­oxy)aceto­yl]hydrazine dihydrate (Zheng *et al.*, 2007 ▸) are 72.7 and 117.6°, respectively, compared to 180.0° in (**I**).

**Figure 2:**
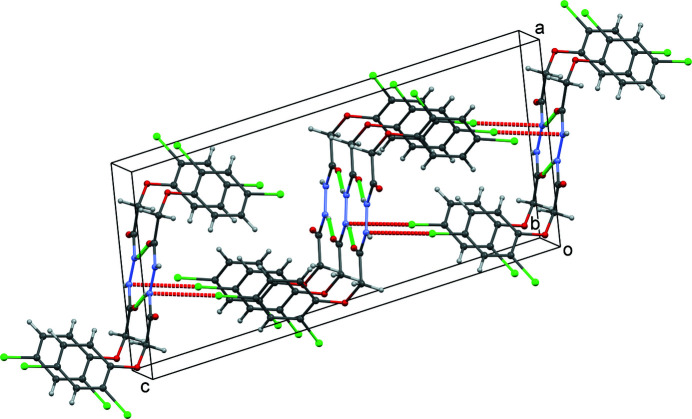
The crystal structure viewed down [010] showing N—H⋯O hydrogen bonds (green dotted lines) and short Cl⋯N inter­actions (red dotted lines).

In the crystal, each mol­ecule is involved in four N—H⋯O hydrogen-bonding contacts, donating two and accepting two bonds, leading to the formation of ribbons propagating parallel to [010] (Table 1 ▸, Fig. 2 ▸). The ribbons are linked by short Cl⋯N contacts perpendicular to the plane of the ribbons and roughly in the *c-*axis direction. The contact involves the *para* Cl atom of the 2,4-di­chloro­phen­oxy group and the nitro­gen atom of the *N*′-acetyl­acetohydrazide group, with a Cl2⋯N1 distance of 3.224 (2) Å (sum of van der Waals’ radii = 3.30 Å).

## Synthesis and crystallization

A mixture of 2-(naphthalen-2-yl­oxy)acetohydrazide (0.47 g, 2.0 mmol) and ethyl 2-cyano-3-eth­oxy­acrylate (0.34 g, 2.0 mmol) in anhydrous ethanol (10 ml) was heated under reflux for 2 h. The solid obtained on cooling was collected by filtration, washed with ethanol, dried, and recrystallized from di­methyl­formamide solution to give colourless plates of (**I**) in 67% yield; m.p. 249–250°C (lit. m.p. 250°C; Abdel-Wahab *et al.*, 2017 ▸).

## Refinement

Crystal data, data collection and structure refinement details are summarized in Table 2 ▸.

## Supplementary Material

Crystal structure: contains datablock(s) I. DOI: 10.1107/S2414314621003187/hb4380sup1.cif

Structure factors: contains datablock(s) I. DOI: 10.1107/S2414314621003187/hb4380Isup2.hkl

Supporting information file. DOI: 10.1107/S2414314621003187/hb4380Isup3.cml

CCDC reference: 2073258

Additional supporting information:  crystallographic information; 3D view; checkCIF report

## Figures and Tables

**Table 1:** Hydrogen-bond geometry (Å, °)

*D*—H⋯*A*	*D*—H	H⋯*A*	*D*⋯*A*	*D*—H⋯*A*
N1—H1⋯O2^i^	0.86	1.94	2.774 (3)	164

Symmetry code: (i) 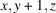 .

**Table 2:** Experimental details

Crystal data	
Chemical formula	C_16_H_12_Cl_4_N_2_O_4_
*M*_r_	438.08
Crystal system, space group	Monoclinic, *P*2_1_/*c*
Temperature (K)	293
*a*, *b*, *c* (Å)	9.7398 (4), 4.6540 (2), 20.1866 (9)
β (°)	100.842 (4)
*V* (Å^3^)	898.71 (7)
*Z*	2
Radiation type	Cu *K*α
μ (mm^−1^)	6.22
Crystal size (mm)	0.16 × 0.10 × 0.01

Data collection	
Diffractometer	Rigaku Oxford Diffraction SuperNova, Dual, Cu at home/near, Atlas
Absorption correction	Gaussian (*CrysAlis PRO*; Rigaku OD, 2015 ▸)
*T*_min_, *T*_max_	0.869, 1.000
No. of measured, independent and observed [*I* > 2σ(*I*)] reflections	5415, 1797, 1504
*R*_int_	0.040
(sin θ/λ)_max_ (Å^−1^)	0.624

Refinement	
*R*[*F* ^2^ > 2σ(*F* ^2^)], *wR*(*F* ^2^), *S*	0.053, 0.153, 1.03
No. of reflections	1797
No. of parameters	118
H-atom treatment	H-atom parameters constrained
Δρ_max_, Δρ_min_ (e Å^−3^)	0.51, −0.42

Computer programs: *CrysAlis PRO* (Rigaku OD, 2015 ▸), *SHELXS* (Sheldrick, 2008 ▸), *SHELXL2018* (Sheldrick, 2015 ▸), *ORTEP-3 for Windows* (Farrugia, 2012 ▸), *Mercury* (Macrae *et al.*, 2020 ▸) and *CHEMDRAW Ultra* (Cambridge Soft, 2016 ▸).

## full crystallographic data

### Crystal data

**Table:**

C_16_H_12_Cl_4_N_2_O_4_	*F*(000) = 444
*M**_r_* = 438.08	*D*_x_ = 1.619 Mg m^−^^3^
Monoclinic, *P*2_1_/*c*	Cu *K*α radiation, λ = 1.54184 Å
*a* = 9.7398 (4) Å	Cell parameters from 2218 reflections
*b* = 4.6540 (2) Å	θ = 4.6–73.9°
*c* = 20.1866 (9) Å	µ = 6.22 mm^−^^1^
β = 100.842 (4)°	*T* = 293 K
*V* = 898.71 (7) Å^3^	Plate, colourless
*Z* = 2	0.16 × 0.10 × 0.01 mm

### Data collection

**Table:**

Rigaku Oxford Diffraction SuperNova, Dual, Cu at home/near, Atlas diffractometer	1504 reflections with *I* > 2σ(*I*)
ω scans	*R*_int_ = 0.040
Absorption correction: gaussian (CrysalisPro; Rigaku OD, 2015)	θ_max_ = 74.2°, θ_min_ = 4.5°
*T*_min_ = 0.869, *T*_max_ = 1.000	*h* = −11→8
5415 measured reflections	*k* = −5→5
1797 independent reflections	*l* = −24→24

### Refinement

**Table:**

Refinement on *F*^2^	0 restraints
Least-squares matrix: full	Hydrogen site location: inferred from neighbouring sites
*R*[*F*^2^ > 2σ(*F*^2^)] = 0.053	H-atom parameters constrained
*wR*(*F*^2^) = 0.153	*w* = 1/[σ^2^(*F*_o_^2^) + (0.0898*P*)^2^ + 0.4345*P*] where *P* = (*F*_o_^2^ + 2*F*_c_^2^)/3
*S* = 1.03	(Δ/σ)_max_ < 0.001
1797 reflections	Δρ_max_ = 0.51 e Å^−^^3^
118 parameters	Δρ_min_ = −0.42 e Å^−^^3^

### Special details

**Table:**

Geometry. All esds (except the esd in the dihedral angle between two l.s. planes) are estimated using the full covariance matrix. The cell esds are taken into account individually in the estimation of esds in distances, angles and torsion angles; correlations between esds in cell parameters are only used when they are defined by crystal symmetry. An approximate (isotropic) treatment of cell esds is used for estimating esds involving l.s. planes.
Refinement. The H atoms were positioned geometrically (N—H = 0.86, C—H = 0.93–0.96 Å) and refined using a riding model with *U*_iso_(H) = 1.2*U*_eq_(C,*N*).

### Fractional atomic coordinates and isotropic or equivalent isotropic displacement parameters (Å^2^)

**Table:**

	*x*	*y*	*z*	*U*_iso_*/*U*_eq_	
C1	0.8216 (2)	0.7834 (5)	0.36947 (12)	0.0365 (5)	
C2	0.8980 (3)	0.5906 (6)	0.33765 (14)	0.0412 (6)	
C3	0.8527 (3)	0.5101 (7)	0.27100 (16)	0.0526 (7)	
H3	0.903880	0.379893	0.250485	0.063*	
C4	0.7314 (3)	0.6258 (8)	0.23597 (15)	0.0557 (7)	
C5	0.6554 (3)	0.8206 (8)	0.26484 (16)	0.0571 (8)	
H5	0.574178	0.899439	0.239873	0.069*	
C6	0.7006 (3)	0.8997 (7)	0.33185 (15)	0.0479 (6)	
H6	0.649162	1.032065	0.351606	0.057*	
C7	0.7949 (3)	1.0250 (5)	0.47047 (14)	0.0381 (6)	
H7A	0.771884	1.200592	0.444919	0.046*	
H7B	0.850851	1.075711	0.513877	0.046*	
C8	0.6610 (2)	0.8789 (5)	0.48107 (12)	0.0333 (5)	
N1	0.5616 (2)	1.0565 (4)	0.49304 (10)	0.0322 (4)	
H1	0.573575	1.239427	0.492170	0.039*	
O1	0.87343 (17)	0.8420 (4)	0.43541 (9)	0.0396 (4)	
O2	0.6471 (2)	0.6192 (4)	0.48048 (13)	0.0548 (6)	
Cl1	1.05074 (8)	0.44859 (19)	0.38265 (4)	0.0618 (3)	
Cl2	0.67176 (10)	0.5161 (3)	0.15306 (4)	0.0858 (4)	

### Atomic displacement parameters (Å^2^)

**Table:**

	*U*^11^	*U*^22^	*U*^33^	*U*^12^	*U*^13^	*U*^23^
C1	0.0307 (11)	0.0369 (12)	0.0445 (12)	−0.0022 (9)	0.0135 (9)	0.0014 (10)
C2	0.0338 (12)	0.0442 (13)	0.0488 (14)	0.0016 (10)	0.0161 (10)	−0.0001 (11)
C3	0.0470 (16)	0.0622 (17)	0.0533 (17)	−0.0043 (13)	0.0219 (13)	−0.0117 (13)
C4	0.0448 (15)	0.077 (2)	0.0469 (14)	−0.0155 (14)	0.0116 (12)	−0.0015 (14)
C5	0.0383 (14)	0.074 (2)	0.0575 (17)	−0.0014 (14)	0.0044 (12)	0.0117 (15)
C6	0.0338 (13)	0.0547 (15)	0.0561 (16)	0.0079 (11)	0.0109 (11)	0.0034 (13)
C7	0.0318 (11)	0.0326 (11)	0.0535 (14)	−0.0028 (9)	0.0172 (10)	−0.0078 (10)
C8	0.0323 (11)	0.0279 (10)	0.0419 (12)	0.0012 (9)	0.0124 (9)	−0.0020 (9)
N1	0.0296 (9)	0.0240 (8)	0.0459 (11)	−0.0006 (7)	0.0142 (8)	−0.0006 (7)
O1	0.0296 (8)	0.0439 (9)	0.0474 (9)	0.0059 (7)	0.0125 (7)	−0.0047 (8)
O2	0.0464 (11)	0.0265 (9)	0.1008 (17)	0.0003 (7)	0.0374 (11)	−0.0041 (9)
Cl1	0.0481 (4)	0.0707 (5)	0.0672 (5)	0.0257 (3)	0.0128 (3)	−0.0051 (4)
Cl2	0.0647 (6)	0.1438 (10)	0.0484 (5)	−0.0318 (6)	0.0094 (4)	−0.0157 (5)

### Geometric parameters (Å, º)

**Table:**

C1—O1	1.359 (3)	C5—H5	0.9300
C1—C6	1.386 (4)	C6—H6	0.9300
C1—C2	1.397 (3)	C7—O1	1.419 (3)
C2—C3	1.386 (4)	C7—C8	1.521 (3)
C2—Cl1	1.723 (3)	C7—H7A	0.9700
C3—C4	1.368 (5)	C7—H7B	0.9700
C3—H3	0.9300	C8—O2	1.216 (3)
C4—C5	1.368 (5)	C8—N1	1.329 (3)
C4—Cl2	1.742 (3)	N1—N1^i^	1.386 (4)
C5—C6	1.391 (4)	N1—H1	0.8600
			
O1—C1—C6	125.4 (2)	C1—C6—H6	119.7
O1—C1—C2	116.6 (2)	C5—C6—H6	119.7
C6—C1—C2	118.0 (2)	O1—C7—C8	111.03 (19)
C3—C2—C1	121.4 (3)	O1—C7—H7A	109.4
C3—C2—Cl1	119.6 (2)	C8—C7—H7A	109.4
C1—C2—Cl1	119.1 (2)	O1—C7—H7B	109.4
C4—C3—C2	118.8 (3)	C8—C7—H7B	109.4
C4—C3—H3	120.6	H7A—C7—H7B	108.0
C2—C3—H3	120.6	O2—C8—N1	122.4 (2)
C3—C4—C5	121.6 (3)	O2—C8—C7	122.6 (2)
C3—C4—Cl2	118.8 (3)	N1—C8—C7	114.89 (19)
C5—C4—Cl2	119.6 (3)	C8—N1—N1^i^	119.3 (2)
C4—C5—C6	119.5 (3)	C8—N1—H1	120.4
C4—C5—H5	120.3	N1^i^—N1—H1	120.4
C6—C5—H5	120.3	C1—O1—C7	118.22 (19)
C1—C6—C5	120.7 (3)		

Symmetry code: (i) −*x*+1, −*y*+2, −*z*+1.

### Hydrogen-bond geometry (Å, º)

**Table:**

*D*—H···*A*	*D*—H	H···*A*	*D*···*A*	*D*—H···*A*
N1—H1···O2^ii^	0.86	1.94	2.774 (3)	164

Symmetry code: (ii) *x*, *y*+1, *z*.
